# Acquisition of Freezing Tolerance of Resurrection Species from Gesneriaceae, a Comparative Study

**DOI:** 10.3390/plants12091893

**Published:** 2023-05-05

**Authors:** Gergana Mihailova, Bekim Gashi, Nikola Krastev, Katya Georgieva

**Affiliations:** 1 Institute of Plant Physiology and Genetics, Bulgarian Academy of Sciences, Acad. G. Bonchev Str., Bl. 21, 1113 Sofia, Bulgaria; gmihailova@bio21.bas.bg (G.M.); nikolakrastev@abv.bg (N.K.); 2 Department of Biology, Faculty of Mathematical and Natural Sciences, University of Prishtina “Hasan Prishtina”, Eqerem Cabej Str No 51, 10020 Prishtina, Kosovo; bekim.gashi@uni-pr.edu

**Keywords:** freezing-induced desiccation, chlorophyll fluorescence, stomatal conductance, pigments, dehydrins, ELIP, *Ramonda serbica*, *Ramonda nathaliae*, *Haberlea rhodopensis*

## Abstract

Resurrection plants have the unique ability to restore normal physiological activity after desiccation to an air-dry state. In addition to their desiccation tolerance, some of them, such as *Haberlea rhodopensis* and *Ramonda myconi*, are also freezing-tolerant species, as they survive subzero temperatures during winter. Here, we compared the response of the photosynthetic apparatus of two other Gesneriaceae species, *Ramonda serbica* and *Ramonda nathaliae*, together with *H. rhodopensis*, to cold and freezing temperatures. The role of some protective proteins in freezing tolerance was also investigated. The water content of leaves was not affected during cold acclimation but exposure of plants to −10 °C induced dehydration of plants. Freezing stress strongly reduced the quantum yield of PSII photochemistry (Y(II)) and stomatal conductance (*g*_s_) on the abaxial leaf side. In addition, the decreased ratio of F_v_/F_m_ suggested photoinhibition or sustained quenching. Freezing-induced desiccation resulted in the inhibition of PSII activity, which was accompanied by increased thermal energy dissipation. In addition, an increase of dehydrins and ELIPs was detected, but the protein pattern differed between species. During recovery, the protein abundance decreased and plants completely recovered their photosynthetic activity. Thus, our results showed that *R. serbica, R. nathaliae,* and *H. rhodopensis* survive freezing stress due to some resurrection-linked traits and confirmed their freezing tolerance.

## 1. Introduction

The rare phenomenon of desiccation tolerance in the vegetative tissues of vascular plants is possessed by so-called resurrection plants. They have the unique ability to restore normal physiological activity after desiccation to an air-dry state [1]. Drought stress inhibits photosynthesis mainly due to stomata closure, thus leading to mesophyll limitations [2] and disruption of the balance of energy capture and utilization via carbon metabolism [3]. As a result, reactive oxygen species (ROS) are generated and oxidative stress occurs in plant cells, leading to serious damage to DNA, proteins, and lipids [4]. Moreover, dehydration causes cell wall shrinkage and following plasma membrane rupture. Thus, resurrection plants have to cope with mechanical, structural, oxidative and metabolic stresses [5]. They evolve different strategies to preserve the integrity of membranes, macromolecules, and photosynthetic machinery by minimizing ROS production in the cells. Downregulation of photosynthesis, enhanced thermal energy dissipation, rearrangement of cell structure, upregulation of antioxidant defense system, synthesis of compatible solutes, and different protective proteins are part of those strategies of plants to cope with drought stress [4,5,6].

In addition to their desiccation tolerance, two of the European resurrection gesneriads, *Haberlea rhodopensis* and *Ramonda myconi*, are also freezing-tolerant species, as they survive subzero temperatures during winter [7,8]. Both plants share the protective strategies used for acquiring desiccation and/or freezing tolerance [9,10,11]. Subzero temperatures are associated with additional complications for plant metabolism—the formation of ice crystals in the apoplast and subsequent disruption of the cell wall and plasma membrane [12]. Our previous investigation showed that temperatures below −6 °C induced desiccation in *H. rhodopensis* plants [8]. 

The protective strategies used by *H. rhodopensis* and *R. myconi* in response to cold acclimation and freezing-induced desiccation include cell ultrastructural changes (chloroplast remodeling, appearance of epidermal channels), downregulation of photosynthesis, enhanced de-epoxidation of the xanthophyll cycle, changes in the abundance of photosynthetic proteins and rearrangement of chlorophyll protein complexes, elevated ratio of unsaturated/saturated fatty acids of membranes, lowered glass transition temperatures, accumulation of different sugars and upregulation of dehydrins and early light-induced protein (ELIP) abundance, upregulation of antioxidant system (phenolics, anthocyanins, enzymatic and nonenzymatic antioxidants, etc.), and elevated levels of polyamines [7,8,10,13,14]. The accumulation of protective substances during dehydration and/or in response to drought or freezing temperatures ensures the successful recovery of resurrection species after rehydration [5,10,15].

*H. rhodopensis* is the second most investigated resurrection species after *Craterostigma plantagineum* [16] and one of few inhabiting the Northern Hemisphere. It is a perennial herbaceous flowering plant, a Tertiary relict on Balkan Peninsula, distributed mainly in the Rhodope Mountains in Bulgaria. *H. rhodopensis* is characterized by high ecological plasticity [17] and its tolerance to desiccation, high light, and high and subzero temperatures has been previously reported [8,18,19,20]. Another two Gesneriaceae resurrection species could be found in the Balkans—*Ramonda serbica* and *Ramonda nathaliae* [21]. The distribution of both species is different—*R. serbica* habitats are mainly situated in Serbia, Albania, Montenegro, North Macedonia, Greece, and Bulgaria, while those of *R. nathaliae* are restricted to North Macedonia, Greece, and Kosovo [21,22]. They grow under different environmental conditions in their natural habitats, as *R. nathaliae* is subjected to harsher climate conditions [22] and is considered to be more resistant to extreme dehydration than *R. serbica* [23]. All three resurrection species are homoiochlorophyllous as they retain their chlorophyll in the course of dehydration [18,23,24]. Rakić et al. [21] suggested that all resurrection gesneriads in Europe are tolerant to subzero temperatures. 

However, the response of *R. serbica* and *R. nathaliae* to freezing stress and the changes at physiological and biochemical levels that help plants to survive harsh winter conditions was not investigated. Thus, we propose that the response of *R. serbica* and *R. nathaliae* to cold and freezing temperatures may differ considering the differences in climatic conditions in their natural habitats. We aimed to investigate the protective mechanisms of plants to cope with low-temperature stress by measuring the changes in photosynthetic activity, dissipation of excitation energy, pigment content, and the abundance of some protective proteins (dehydrins, ELIPs). In addition, we compared the response to cold acclimation and freezing stress of two *Ramonda* species, to that of the freezing-tolerant *H. rhodopensis*. The experiment was performed in ex situ environmental conditions from November (cold acclimation) to June (full recovery). 

## 2. Results

### 2.1. Photosynthetic Activity during Cold Acclimation

**Figure 1:**
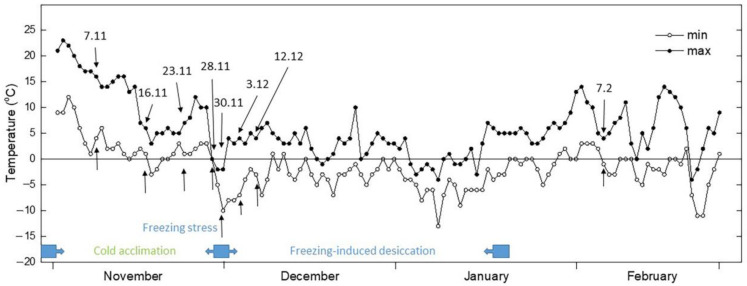
Changes of minimal and maximal daily temperatures (from November to February) during the autumn/winter season of 2018–2019. The numbers and arrows indicate the dates of measurements and sampling during ex situ environmental conditions.

**Figure 2:**
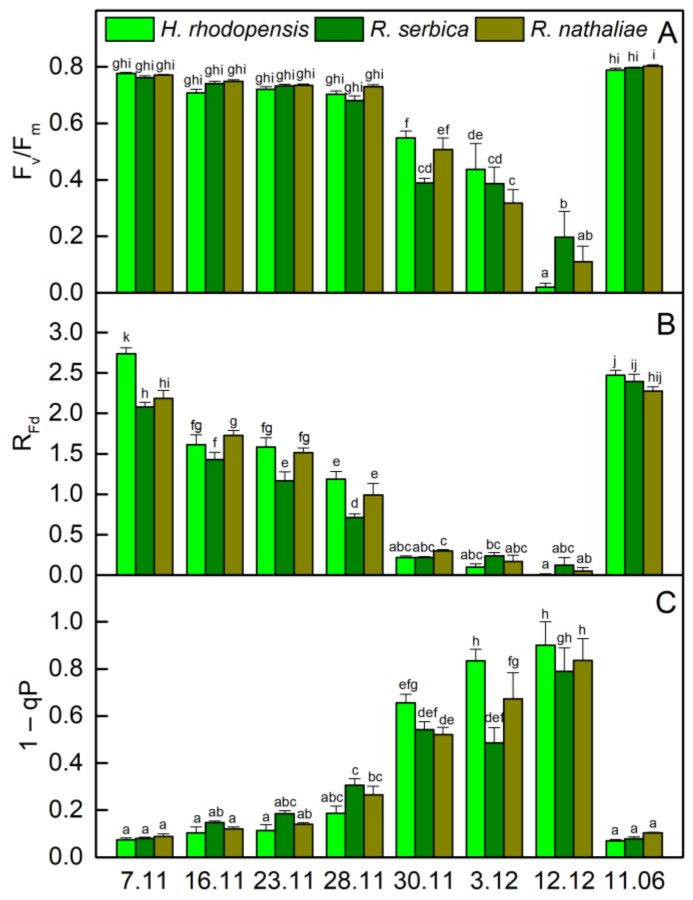
Maximal quantum efficiency (F_v_/F_m_; (**A**)), vitality index (R_Fd_; (**B**)) and excitation pressure (1 − qP; (**C**)) of *H. rhodopensis*, *R. serbica*, and *R. nathaliae* leaves during cold acclimation (7–28 November), freezing stress (30 November), freezing-induced desiccation (3 December–7 February) as well after recovery of plants (11 June) in ex situ environmental conditions. Values are given as mean ± SE. Data represent the mean of *n* = 6. Changes between plants were statistically compared. The same letters within a graph indicate no significant differences assessed by the Fisher LSD test (*p* ≤ 0.05) after performing ANOVA.

**Figure 3:**
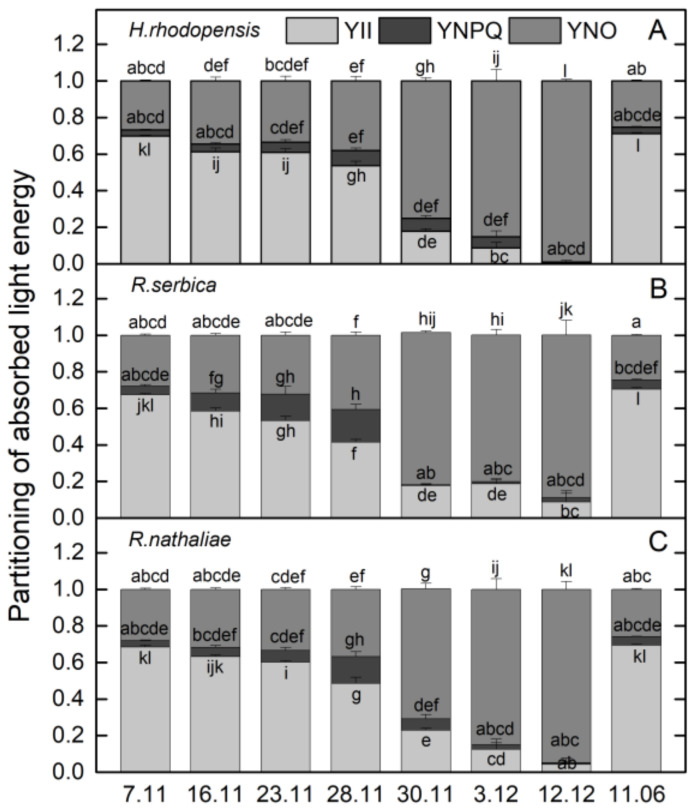
Changes in the quantum yield of PSII electron transport during illumination, Y(II), the quantum yield of light-induced non-photochemical fluorescence quenching, (Y)NPQ, and the quantum yield of non-regulated heat dissipation and fluorescence emission, Y(NO), in *H. rhodopensis* (**A**), *R. serbica* (**B**), and *R. nathaliae* (**C**) leaves during cold acclimation (7–28 November), freezing stress (30 November), freezing-induced desiccation (3 December–7 February) as well after recovery of plants (11 June) in ex situ environmental conditions. Values are given as mean ± SE. Data represent the mean of *n* = 6. Changes between plants were statistically compared. The same letters within a graph indicate no significant differences assessed by the Fisher LSD test (*p* ≤ 0.05) after performing ANOVA.

The temperatures gradually decreased in November and the average day/night temperature until 28 November was 11/2 °C (Figure 1). Exposure of plants to low positive temperatures is considered cold acclimation (CA). The appearance of *H. rhodopensis*, *R. serbica,* and *R. nathaliae* is presented in Figure S1. The results showed that the maximum efficiency of PSII, (F_v_/F_m)_, was not affected during the CA of *H. rhodopensis* and *Ramonda* species studied (Figure 2A). In fact, the RWC of leaves was about 80–85% (Table 1) and the main part of absorbed light energy was used for photochemistry (Figure 3A). However, in contrast to F_v_/F_m_, the actual efficiency of PSII, Y(II) significantly decreased during CA and it was reduced by 23%, 40%, and 30% in *H. rhodopensis*, *R. serbica,* and *R. nathaliae*, respectively on the 28 November (Figure 3A).

**Figure 4:**
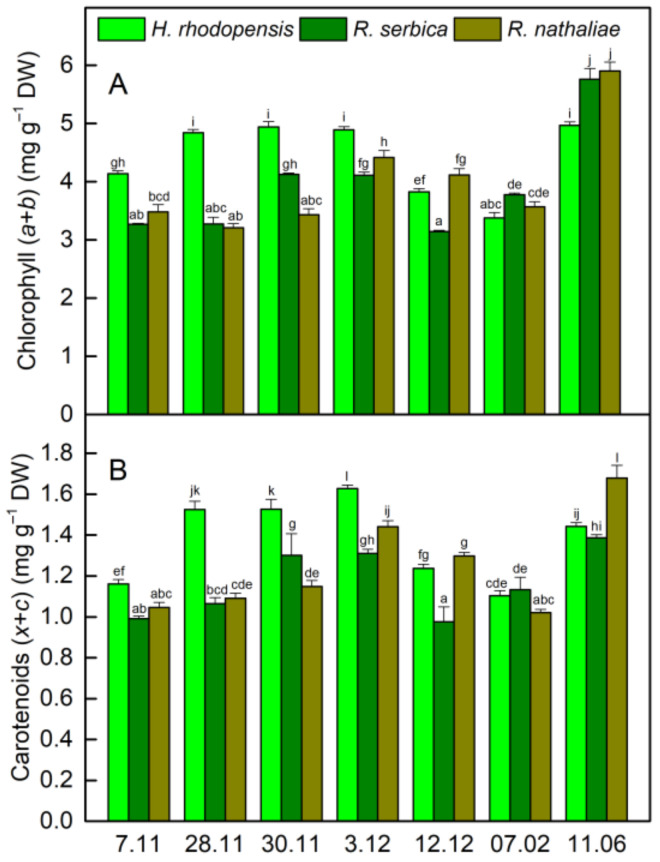
Alterations in chlorophyl (**A**) and carotenoid (**B**) content of *H. rhodopensis*, *R. serbica,* and *R. nathaliae* leaves during cold acclimation (7–28 November), freezing stress (30 November), freezing-induced desiccation (3 December–7 February) as well after recovery of plants (11 June) in ex situ environmental conditions. Values are given as mean ± SE. Data represent the mean of *n* = 8. Changes between plants were statistically compared. The same letters within a graph indicate no significant differences assessed by the Fisher LSD test (*p* ≤ 0.05) after performing ANOVA.

**Figure 5:**
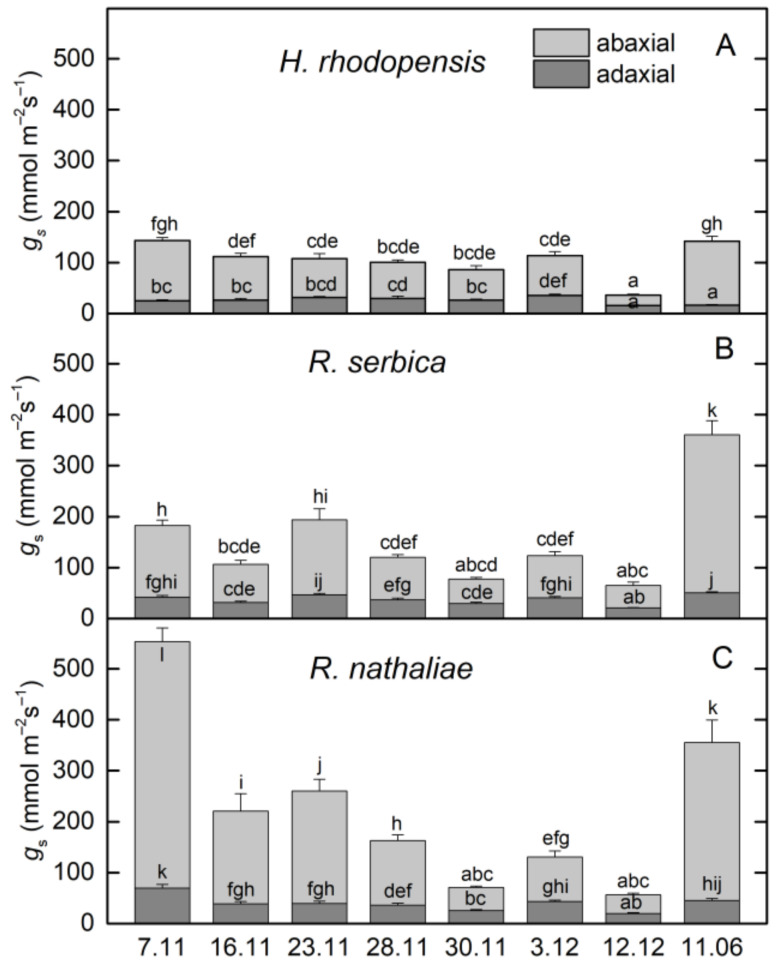
Changes in stomatal conductance (*g*_s_) of abaxial (light gray) and adaxial (dark gray) leaf surface of *H*. *rhodopensis* (**A**), *R. serbica* (**B**), and *R. nathaliae* (**C**) during exposure to cold acclimation (7–28 November), freezing stress (30 November), freezing-induced desiccation (3 December–7 February) as well after recovery of plants (11 June) in ex situ environmental conditions. Values are given as mean ± SE. Data represent the mean of *n* = 6; Changes between plants were statistically compared. The same letters within a graph indicate no significant differences assessed by Fisher’s LSD test (*p* ≤ 0.05) after performing ANOVA.

The changes in the ratio of chlorophyll (Chl) fluorescence decrease (F_m_ − F_s_) to the steady-state Chl fluorescence (F_s_), R_Fd_ permits a fast evaluation of the photosynthetic activity and has been shown to correlate with the CO_2_ assimilation rate of leaves [25]. R_Fd_ values strongly declined during CA and they were reduced by about 60% in three Gesneriaceae species (Figure 2B). The high correlation coefficient of Pearson (*r* > 0.970) was determined for the changes in R_Fd_ and Y(II), but R_Fd_ was the most sensitive parameter to low temperatures. It should be mentioned that the decrease in the photosynthetic activity during CA was not due to changes in Chl content (Figure 4A). The Chl (*a* + *b*) content of both *Ramonda* species was not significantly influenced until 28 November and some enhancement in its content was measured in *H. rhodopensis* leaves. In addition, a good correlation was observed between the changes in R_Fd_ and stomatal conductance (*g*_s_), measured on the abaxial leaf side (*r* > 0.820). Similar to our previous results on *H. rhodopensis*, we registered much higher *g*_s_ values on the abaxial side of *R. serbica* and *R. nathaliae* leaves compared to the adaxial side (Figure 5). The reduction in the photochemical activity of PSII during CA was accompanied by an increased amount of closed PSII reaction centers, estimated by the changes in 1 − qP or so-called excitation pressure (Figure 2C). High negative correlation coefficient of Pearson (*r* > −0.940) was determined for the changes in Y(II) and 1 − qP. Excitation pressure is suggested to be a major prerequisite for inducing efficient dissipation of excess excitation energy, thereby protecting the PSII reaction center from overexcitation. The results on the partitioning of absorbed light clearly showed that both the quantum yield of light-induced non-photochemical fluorescence quenching, Y(NPQ), and the quantum yield of non-regulated heat dissipation and fluorescence emission, Y(NO) increased during CA (Figure 3).

### 2.2. Effect of Freezing Stress on Photosynthetic Efficiency

Exposure of plants to −10 °C (30 November) decreased the leaf RWC, being the strongest in *H. rhodopensis* (by 32%) and weakest in *R. nathaliae* (by 16%). Freezing stress led to a strong reduction in photosynthetic efficiency. The ratio F_v_/F_m_ decreased by 29%, 49%, and 34% in *H. rhodopensis*, *R. serbica,* and R. *nathaliae* (Figure 2A), respectively, whereas Y(II) was reduced by more than 70% (Figure 3) compared with those measured on 7 November; *g*_s_ additionally declined (Figure 5) and R_Fd_ was strongly inhibited (Figure 2B). In addition, the values of 1− qP increased more than 6 times (Figure 2C). Enhanced excitation pressure was accompanied by a double increase in Y(NO) (Figure 3) mainly due to a significant reduction in F_m_ (Figure S2). On the other hand, freezing stress caused a decrease in Y(NPQ) values, which was weaker in *H. rhodopensis* and stronger in *R. serbica*. 

Fast desiccation of plants was induced when the temperature declined to −10 °C (30 November). Their RWC was reduced to 50% on 3 December and reached about 20% on 12 December (Table 1). During freezing-induced desiccation, the photosynthetic activity was strongly inhibited resulting in suppressed Y(NPQ), and the main part of absorbed light energy was emitted as heat. In fact, the proportion of Y(NO) was more than 90% in desiccated leaves (Figure 3), when the 1 − qP reached the highest values (Figure 2C). In addition to increased harmless dissipation of excess excitation energy that cannot be used for photochemistry, the carotenoid content increased by 40% at the beginning of desiccation (date 3 December) and was closed to that measured on 7 November in desiccated plants (7 February). 

As might be expected for resurrection plants, their photosynthetic activity was fully restored after rehydration.

### 2.3. Protective Protein Accumulation during Cold Acclimation and Freezing-Induced Desiccation

#### 2.3.1. Dehydrins

**Figure 6:**
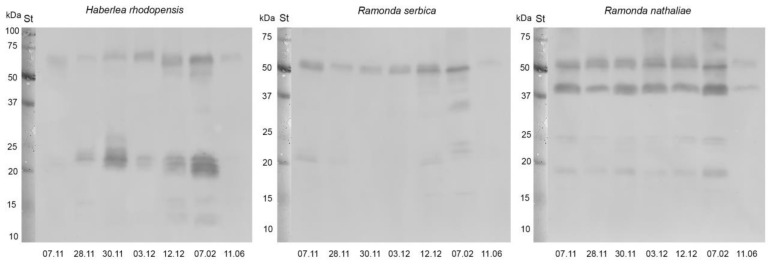
Western blots of dehydrins in leaves of *H. rhodopensis*, *R. serbica,* and *R. nathaliae* during cold acclimation (7–28 November), freezing stress (30 November), freezing-induced desiccation (3 December–7 February) as well after full recovery (11 June) of plants. 30 μg protein was applied per lane. St: Precision Plus Dual Color Protein™ Prestained Standards (Bio-Rad, Hercules, CA, USA).

Dehydrin abundance in the leaves of *H. rhodopensis*, *R. serbica,* and *R. nathaliae* during CA and freezing-induced desiccation was monitored by Western blot using specific antibodies raised against the conserved K-segment of the proteins. Dehydrins were present in all investigated samples and at least 10 dehydrin bands with apparent molecular weight between 65 to 12 kDa were detected, but the protein pattern differed among the three species—*H. rhodopensis* and *R. serbica* profiles were more similar, while that of *R. nathaliae* was distinct (Figure 6). Cold acclimation (28 November), freezing stress (30 November), and freezing-induced desiccation (date 3 December–7 February) increased the content of the proteins and these changes were most pronounced in the leaves of *H. rhodopensis*. Minor changes in dehydrin content were detected in *R. serbica*. The bands around 65 kDa and 20–22 kDa in *H. rhodopensis*, around 52 kDa in *R. serbica,* and around 52 kDa and 38 kDa in *R. nathaliae*, were more pronounced. The band detected around 38 kDa was only present in *R. nathaliae*. Some shift of the molecular weight of dehydrins in the upper bands (around 60–65 kDa in *H. rhodopensis* and 52 kDa in *Ramonda* species) could be seen during cold acclimation and freezing-induced desiccation in all three plants. The dehydrin abundance decreased after the recovery (11 June) of the investigated species. The signals were faint as the band around 65 kDa was the only one presented in *H. rhodopensis*, the 52 kDa band in *R. serbica*, and two bands, around 38 and 52 kDa, in *R. nathaliae*. 

#### 2.3.2. Early Light-Induced Proteins (ELIPs)

**Figure 7:**
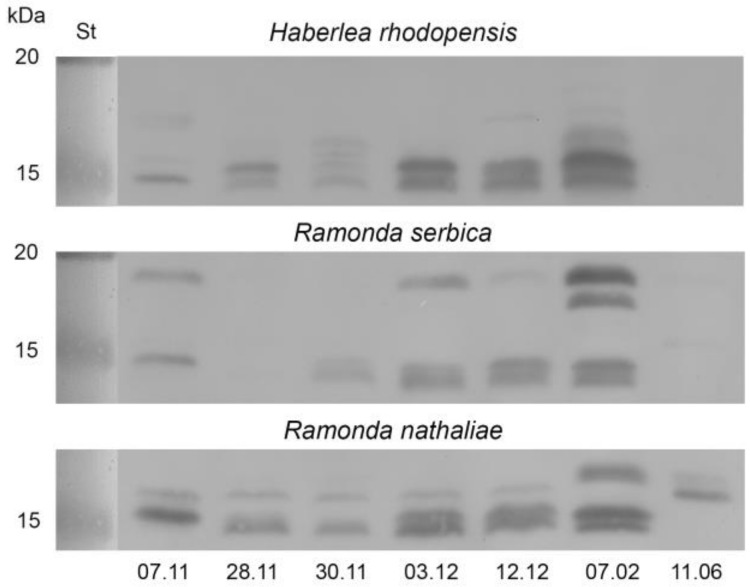
Western blots of ELIPs in leaves of *H. rhodopensis*, *R. serbica,* and *R. nathaliae* during cold acclimation (7–28 November), freezing stress (30 November), and freezing-induced desiccation (3 December–7 February) as well after full recovery (11 June) of plants. Thirty μg of protein was applied per lane. St: Precision Plus Dual Color Protein™ Prestained Standards (Bio-Rad, Hercules, CA, USA).

As well as dehydrins, we monitored the accumulation of ELIPs in the leaves of the three resurrection species during cold acclimation and freezing-induced desiccation by Western blot. We detected two major and several minor bands in almost all samples with apparent molecular weight between 14–19 kDa, 13–19 kDa, and 14–17 kDa in *H. rhodopensis*, *R. serbica,* and *R. nathaliae*, respectively (Figure 7). The protein pattern was very similar in all three species. The two major bands are positioned close to 15 kDa and ELIPs were presented in well-hydrated plants with high physiological activity on 7 November. Cold acclimation (28 November) did not change significantly the ELIP abundance in *H. rhodopensis* and *R. nathaliae*, while in *R. serbica* its content surprisingly decreased. Temperatures drop to −10 °C (30 November) did not affect ELIP abundance in *R. nathaliae*, while in *H. rhodopensis* and *R. serbica* new bands could be distinguished. Freezing-induced desiccation (3 December–7 February) increased ELIP content in all resurrection species and in fully desiccated plants new ELIP bands appeared. In the leaves of *R. serbica* and *R. nathaliae*, two of the newly expressed bands, around 17 and 17–19 kDa respectively, were characterized by very high abundance. In *H. rhodopensis* those bands were very weak. During the recovery (date 11 June) of *H. rhodopensis* and *R. serbica,* ELIP signals almost disappeared, while in *R. nathaliae* leaves two bands could be clearly detected.

## 3. Discussion

### 3.1. Physiological Changes in Response to Cold Acclimation and Freezing-Induced Desiccation

The resurrection plants growing in mountainous areas are exposed to and must overcome various stress factors. Many studies have been performed on the desiccation tolerance of *H. rhodopensis*, *R. serbica,* and *R. nathaliae*; however, the investigations on their freezing tolerance are restricted only to *H. rhodopensis* [8,10,14]. In the present study, we explored the response of these endemic and relict plants from the Balkan Peninsula to low positive and subzero temperatures. Exposure of plants to freezing stress induced dehydration of plants and they survived the harsh conditions due to some resurrection-linked traits. Being homoiochlorophyllous, they retain the chlorophyll content and photosynthetic apparatus during desiccation, which allow their rapid recovery upon rehydration. But on the other hand, the high amount of Chl molecules could be a source of singlet oxygen production and oxidative damage. We did not determine significant changes in Chl content in both *Ramonda* species during exposure to low temperatures and following desiccation of plants (Figure 4A). It decreased by about 18% only after prolonged exposure of dry *H. rhodopensis* leaves to low temperatures, which is in agreement with the changes in Chl content induced by drought stress [18,26]. In addition, the reduction of total chlorophyll content in desiccated *R. serbica* and *R. nathaliae* leaves about 15–21% and 11–13%, respectively, was reported [23,27]. For example, changes in Chl content were determined upon drying *R. serbica* from negligible [24] to 40% [28], which is most probably due to differences in the experimental conditions (light, temperature, humidity). Moreover, decline of δ-aminolevulinic acid dehydratase activity, which is part of the biosynthetic pathway of Chl, was reported in both *Ramonda* species during dehydration [29].

In addition to the upregulation of the antioxidant system, resurrection plants avoid oxidative stress during dehydration by leaf folding so that the hairy abaxial leaf side becomes exposed to the light and there is a strong reduction in leaf area (Figure S1) and the downregulation of photosynthesis (Figure 2 and Figure 3). Reversible leaf curling was observed during CA when the overnight temperature decreased to −2 °C. Despite the unchanged RWC during CA, the efficiency of PSII electron transport gradually decreases (Figure 3) and it was significantly reduced before exposure of plants to −10 °C. Taking into account the linear correlation between changes in R_Fd_ and CO_2_ assimilation previously observed [25], the higher sensitivity of R_Fd_ than Y(II) suggested that CO_2_ assimilation was more sensitive to low temperatures. Similarly, it has been shown that the reduction of CO_2_ assimilation was affected before photochemical activity under moderate water stress conditions [18,28]. Formerly, we have shown that the amount of both large and small subunits of the Calvin cycle protein Rubisco, RbcL, and RbcS, decreased as a result of cold and freezing temperatures and freezing-induced desiccation [8]. Thus, the higher decrease in CO_2_ assimilation than electron transport with low temperatures may also be due to the effect of temperature on the activity of enzymes involved in CO_2_ fixation. A significant reduction of *g*_s_ measured on the abaxial leaf side was observed during CA of three Gesneriaceae species (Figure 5). Interestingly, the values of *g*_s_ measured in *R. nathaliae* on 7 November were much higher compared with *H. rhodopensis* and *R. serbica.* It could be related to the higher frequency of stomata observed in *R. nathaliae* than in *R. serbica* and their distribution on both sides of the leaves [27]. Comparison of photosynthetic activity of three resurrection species in CA state showed that low positive temperatures decreased the quantum efficiency PSII electron transport, Y(II), and R_Fd_ in *R. serbica* more compared with *H. rhodopensis* and *R. nathaliae* (23–28 November, Figure 2 and Figure 3). A higher sensitivity of photosynthetic activity in *R. serbica* compared with *R. nathaliae* to high temperatures (up to 40 °C) and during water deficit has been reported [23,27] and this was related to drier and warmer habitats of *R. nathaliae* [22].

The response of three desiccation-tolerant species to freezing stress was similar. Exposure of plants to −10 °C decreased their RWC and the inhibition of photosynthetic activity was close to that of drought-induced desiccation. Freezing stress led to photoinhibition of PSII as assessed by the decrease in F_v_/F_m_. The reduced F_v_/F_m_ ratio was due to a drop in F_0_ values and a particularly strong decrease in F_m_ (Figure S2). The most significant decline in F_v_/F_m_ was observed in *R. serbica*. The significant inhibition of F_v_/F_m_ during freezing-induced desiccation was mostly attributable to the strong decrease in F_m_ accompanied by a moderate decrease in F_0_. Indeed, a high Pearson correlation coefficient was determined for the changes in Fm and F_v_/F_m_ (*r* > 0.96). The decrease in F_0_ indicates the transformation of PSII centers to highly efficient quenchers, converting excitation energy to heat [30], while photoinhibition or maintaining a high NPQ in the dark, as observed in wintering evergreens [31] can lead to decreased F_m_ values. Our results indicate that the reduced Y(II), when RWC drop to about 20 % (12 December), was mainly due to a decrease in the proportion of open PSII centers (qP) as well as a decrease in their excitation capture efficiency (F_v_′/F_m_′; Figure S3). Downregulation of photosynthesis of overwintering plants was considered an adaptive photoprotective strategy that lowers photosynthetic efficiency to prevent damage [32,33]. 

All changes in photosynthetic activity induced by freezing stress were reparable due to the involvement of defense mechanisms, such as high amounts of carotenoids and increased dissipation of excess excitation energy that cannot be used in photosynthesis. The carotenoid content increased during CA, reaching a maximum after exposure of plants to −10 °C and, despite some decline, it remained high during freezing-induced desiccation (Figure 4). Similarly, it has been shown that carotenoid content was not significantly changed in desiccated leaves of *R. serbica* and *R. nathaliae* [27,28]. 

The downregulation of the photosynthetic electron transport rate was accompanied by a significant enhancement in excitation pressure (Figure 2) and enhancement in thermal energy dissipation (Figure 3). Our results showed that non-photochemical fluorescence quenching has a major role in preventing photoinhibition during CA. Y(NPQ) gradually increased during exposure of plants to low temperatures and was maximal in the CA state (28 November). It remained high in *H. rhodopensis* and *R. nathaliae* when the temperature dropped to −10 °C. NPQ requires a trans-thylakoid pH, the presence of zeaxanthin and conformational changes in the light-harvesting antenna [24,34,35]. Fernández-Marín et al. [7] showed that freezing can induce zeaxanthin synthesis even in the absence of light and the potential activity of the enzyme violaxanthin de-epoxidase occurred at −7 °C. During freezing-induced desiccation, when photosynthesis was significantly inhibited, a low rate of electron transport induced a decreased ΔpH across the thylakoid membranes, and the main part of excess excitation energy was dissipated as heat. In severely desiccated plants Y(NO) became the main mechanism of energy dissipation providing effective protection against overexcitation. 

### 3.2. The Role of Protective Proteins for Acquisition of Freezing Tolerance

Vitrification of the subcellular milieu during drought- or freezing-induced desiccation strongly impacts the biochemical processes in the cells. Enhanced cumulation of stress-induced proteins, such as dehydrins and ELIPs, is one of the main strategies used by resurrection plants to protect the macromolecules in the cells from the detrimental effect of desiccation [5]. Dehydrins belong to group 2 of LEA proteins and they are disordered hydrophilic proteins that are highly expressed under drought and cold stresses [36,37]. Our results revealed the different protein pattern accumulation of dehydrins in *H. rhodopensis*, *R. serbica,* and *R. nathaliae* during cold acclimation and freezing-induced desiccation as that of *R. nathaliae* differed from that of the other two plants. Immunoblot signals showed that dehydrin expression could be constitutive and/or induced in the three investigated species. Accumulation of dehydrins around 24–28 kDa was monitored in both *Ramonda* plants under freezing-induced desiccation, while in *H. rhodopensis* the proteins the molecular weight was around 20–22 kDa. Similar results regarding *H. rhodopensis* were obtained in our previous study [10]. In the protein pattern of *R. nathaliae* could be distinguished a unique major band around 38 kDa in all samples investigated was not present in *R. serbica* and *H. rhodopensis* leaves. Four upregulated dehydrin bands between 24–28 kDa were monitored in desiccated leaves of *R. nathaliae* [38]. The close molecular weight of detected proteins could be a result of phosphorylation as posttranslational modification [39] or alternative start and stop codons [40] thus generating different proteins from one gene. Low molecular weight dehydrins (20 and 12 kDa) accumulated during desiccation and after recovery in *H. rhodopensis* thylakoids [10,41]. Although the exact physiological functions of dehydrins have not yet been elucidated, it was suggested that they play a crucial role in the acquisition of desiccation tolerance stabilizing cell macromolecules, and acting as ROS scavengers [42]. Recently, LEA proteins in *R. serbica* leaves were characterized [43]. Dehydrins were divided into two groups, DEH1 and DEH2, and they are characterized by a very highly disordered structure with a typical random coil conformation. More members of DEH1 were upregulated during desiccation compared to members of DEH2. In addition, the structure of K-, S-, and Y-segments were elucidated. The authors suggested a nuclear localization for some of the dehydrins identified. 

Van Buren et al. [44] proposed that duplication of ELIP genes in the genomes of resurrection plants is related to the evolution of desiccation tolerance and the protection of chloroplasts from photooxidative damage. ELIP transcripts and proteins are highly expressed in the leaves of *H. rhodopensis* in response to desiccation and during the early hours after rehydration [10,39,41,45,46,47]. Recently, we found that rehydration after freezing-induced desiccation enhanced the ELIP transcript abundance more strongly in leaves compared with rehydration after drought [41]. Our results suggest that ELIPs play a role in the acquisition of freezing tolerance in *R. serbica* and *R. nathaliae*. To our knowledge, this is the first time when ELIP proteins are reported in *R. serbica* and *R. nathaliae*. The highest ELIP abundance matched the lowest levels of PSII efficiency. It was suggested that ELIPs stabilize photosynthetic complexes via chlorophyll binding [48]. 200 tandemly duplicated ELIP genes were determined in the genome of *C. plantagineum* [49]. All three investigated resurrection species *R. serbica*, *R. nathaliae,* and *H. rhodopensis*, showed different protein profiles of ELIPs accumulation during cold acclimation and freezing-induced desiccation. This could be related to the different environmental conditions in their natural habitats and/or gene duplications. The ELIP protein pattern of *H. rhodopensis* is very similar to previously obtained results [10]. Interestingly, *H. rhodopensis* plants rehydrated after drought- and freezing-induced desiccation also showed different protein patterns of ELIP accumulation [41]. Recovery decreased the ELIP content in *R. serbica* and *H. rhodopensis*, and to a lesser extent in *R. nathaliae*. Although the pattern of ELIP accumulation is similar in the three plants, it was definitely species-specific. 

## 4. Materials and Methods

### 4.1. Plant Material and Experimental Design

*Haberlea rhodopensis* Frivaldszky tufts were initially collected from the Rhodope Mountains and reproduced vegetatively under ex situ condition as previously described, *Ramonda serbica* Pančić plants were collected from the Sharri Moutains, near the city of Prizren (Kosovo), and *Ramonda nathaliae* Pančić & Petrović were collected from Glloboçica (Kosovo), near the border with North Macedonia. The plants were cultivated under ex situ environmental conditions. Cold acclimation and freezing tolerance were studied under ex situ environmental conditions in Sofia, Bulgaria. The samples were collected at different time points from November 2018 to June 2019, during cold acclimation in November (7–28 November), after exposure to freezing stress (30 November), during freezing-induced desiccation (3 December–7 February) and after recovery of plants in June. Minimum and maximum daily ambient temperatures were recorded from November until June. Over the autumn–winter period, the average daily light intensity under natural conditions ranged between 30–60 μmol m^−2^ s^−1^ PPFD. At each time point, leaves were collected from at least five different tufts (with 3–7 individual plants in a tuft) to have mean samples. 

### 4.2. Determination of RWC

The RWC of leaves was determined gravimetrically by weighing them before and after oven-drying at 80 °C to a constant mass and expressed as a percentage of water content in dehydrated tissue compared with water-saturated tissues using the following equation: RWC (%) = (FW − DW)/(TW − DW) × 100, where FW—fresh weight, DW—dry weight, and TW—turgid weight. TW was measured on leaves maintained for 12–16 h at 4 °C in the dark floating on water.

### 4.3. Pigment Content Determination

One-hundred mg of leaves was homogenized with ten mL of 80% acetone and the homogenate was centrifuged at 5000× *g* for 20 min at 4 °C. The Chl *a*, Chl *b,* and total carotenoid contents were determined spectrophotometrically by measuring the absorbance at 663, 645, and 460 nm using Multiskan Spectrum (Thermo Fisher Scientific, Waltham, MA, USA). The pigment content was calculated according to the equations of Lichtenthaler [50]. The data were presented on a dry weight basis.

### 4.4. Stomatal Conductance

Leaf porometer SC-1 (Decagon Devices, Inc., Pullman, WA, USA) equipped with a desiccant chamber was used to measure the stomatal conductance on the adaxial and abaxial side of the leaves. To ensure accurate conductance reading the sensor head was calibrated before measurements. Measurements were always performed between 11 a.m. and 1 p.m. Six leaves were marked and measured throughout the experiment.

### 4.5. Chlorophyll a Fluorescence Induction

Chl *a* fluorescence induction was measured with a portable fluorometer *PAM-2500* (Heinz Walz GmbH, Effeltrich, Germany). The leaves were dark-adapted for 15 min and PAR of 90 μmol (photon) m^−2^ s^−1^ was used for the measurements. All used basic parameters were given by *PamWin-3* software (Heinz Walz GmbH, Effeltrich, Germany). The maximum efficiency of PSII photochemistry was calculated as F_v_/F_m_ immediately after the predarkening period. The actual efficiency of PSII electron transport during illumination was estimated at a steady state as Y(II) = (F_m_′ − F_s_)/F_m_′ [51]. The quantum yield of light-induced non-photochemical fluorescence quenching was calculated as (Y)NPQ = (F_m_/F_m_’) − 1 and the quantum yield of non-regulated heat dissipation and fluorescence emission as Y(NO) = F_s_/F_m_ [52]. All three yield parameters sum up to 1: Y(II) + Y(NPQ) + Y(NO) = 1. The ratio of the chlorophyll fluorescence decrease to steady-state fluorescence (used as a vitality index) was calculated as R_Fd_ = F_d_/F_s_, where F_d_ = F_m_ − F_s_ [25]. The excitation pressure of PSII, which gives an approximate measure of the reduction state of the first electron acceptor Q_A_ of PSII, was calculated as 1 − qP, as qP is determined by the equation qP = (F_m_′ − Fs)/(F_m_′ − F_0_) [53]. Six leaves from every plant species were marked and measured throughout the experiment. 

### 4.6. Total Leaf Protein Extraction, SDS-PAGE, and Western Blot Analysis

Total leaf proteins were extracted in the sample buffer as described by Mihailova et al. [8]. The protein content was determined according to Bradford [54]. Isolated samples were separated on 12% (dehydrins) or 16% (ELIPs) SDS-PAGE (SE260 Mighty Small II, Hoefer, Holliston, MA, USA) according to Laemmli [55], modified by adding 8.0% glycerol to stacking and separating gels using a constant current of 20 mA per gel. Each lane contains 30 μg total leaf protein. Using semi-dry transfer (TE70X, Hoefer, Holliston, MA, USA) the proteins were blotted on nitrocellulose membrane for 90 min at a current of 1 mA cm^−2^. Prestained protein standard (Precision Plus Protein™ Dual Color Standards, Bio-Rad, Hercules, CA, USA) was used for monitoring electrophoresis separation and transfer efficiency. Blots were probed with primary antibodies against ELIPs (AS06 147A, Agrisera, Vännäs, Sweden) and dehydrin K-segment (AS07 206A, Agrisera, Vännäs, Sweden). Horseradish peroxidase-conjugated goat anti-rabbit secondary antibody was used (AS09 602, Agrisera, Vännäs, Sweden). The resulting bands were visualized by chemiluminescence, and signals were recorded on X-ray Blue films (Carestream Dental LLC, Atlanta, GA, USA). Films were scanned using an Epson Perfection V850 PRO scanner (Seiko Epson Corporation, Suwa, Japan).

### 4.7. Statistical Analysis

Leaves for biochemical analysis were sampled from five different tufts at each time point. Total leaf proteins were isolated two times and SDS–PAGE and immunoblot analysis were repeated twice. For Chl *a* fluorescence induction and stomatal conductance, the same six leaves were measured during the experiments. Comparison of means was made by the Fisher least significant difference (LSD) test at *p* ≤ 0.05 following ANOVA. A statistical software package (StatGraphics Plus, version 5.1 for Windows, The Plains, VA, USA) was used.

Pearson’s correlation coefficient (*r*) was used to measure the strength of a linear association between two variables. 

## 5. Conclusions

A comparison of the results obtained upon CA revealed the high resistance of investigated resurrection plants to low positive temperatures. They were able to maintain high photosynthetic activity and the main part of excitation energy was used for photochemistry. Plants keep their RWC during CA; thus, fresh leaves were exposed to subzero temperatures. Exposure of plants to −10 °C induced desiccation of plants and they survived the harsh conditions due to some resurrection-linked traits. Downregulation of photosynthesis, increased carotenoid content, and presumably enhanced dissipation of excess excitation energy protect plants against photooxidation. The protection of PSII during CA was mainly due to significant enhancement of non-photochemical quenching, whereas during freezing-induced desiccation the main part of absorbed light energy was emitted as thermal energy dissipation. Similar to *H. rhodopensis*, the accumulation of stress-induced proteins, such as dehydrins and ELIPs, suggests a role in the acquisition of freezing tolerance in *R. serbica* and *R. nathaliae*. Differences in the protein profile of ELIPs and dehydrins accumulation during cold acclimation and freezing-induced desiccation were observed between the three plant species. Regarding the photosynthetic activity, a stronger reduction in the quantum efficiency of PSII electron transport in *R. serbica* compared with *R. nathaliae* and *H. rhodopensis* was found only in the CA state, while the response of three desiccation-tolerant species to freezing stress was similar. 

## Figures and Tables

**Table 1:** Relative water content (RWC) of the leaves of *H. rhodopensis*, *R. serbica,* and *R. nathaliae* during cold acclimation (7–28 November), freezing stress (30 November), freezing-induced desiccation (3 December–7 February) as well after recovery of plants (11 June) in ex situ environmental conditions. Values are given as mean ± SE.

RWC (%)			
Date	*H. rhodopensis*	*R. serbica*	*R. nathaliae*
7 November	80.1 ± 0.8	85.5 ± 1.7	85.4 ± 2.6
28 November	80.6 ± 2.3	80.5 ± 3.2	85.1 ± 5.5
30 November	67.5 ± 4.1	76.9 ± 4.6	84.3 ± 5.0
3 December	50.5 ± 1.4	56.4 ± 4.0	53.9 ± 7.0
12 December	18.1 ± 0.5	18.9 ± 0.6	29.2 ± 1.0
7 February	7.0 ± 1.5	12.6 ± 1.6	8.9 ± 1.4
11 June	77.4 ± 1.9	84.1 ± 2.0	83.4 ± 1.6

## Data Availability

The data is contained within the article and Supplementary Materials.

## Supplementary Materials

The following supporting information can be downloaded at: https://www.mdpi.com/article/10.3390/plants12091893/s1, Figure S1: Control, desiccated by freezing temperatures, recovered plants as well as a close-up view of H. rhodopensis, R. serbica, and R. nathaliae flowers. Figure S2: Minimum and maximum fluorescence levels of H. rhodopensis, R. serbica, and R. nathaliae leaves during cold acclimation, freezing stress, freezing-induced desiccation as well after recovery of plants in ex situ environmental conditions. Figure S3: Changes of the open PSII centers and thermal energy dissipation of H. rhodopensis, R. serbica, and R. nathaliae leaves during cold acclimation, freezing stress, freezing-induced desiccation as well after recovery of plants in ex situ environmental conditions.
