# Supplementary material for: Acquisition of Freezing Tolerance of Resurrection Species from Gesneriaceae, a Comparative Study
Source: Plants (Basel). 2023 May 5;12(9):1893. doi: 10.3390/plants12091893 (PMC10180725; doi:10.3390/plants12091893)
Supplement: Supplementary file 1 [file plants-12-01893-s001.zip › plants-2342466-supplementary.pdf]

plants

Supplementary Materials

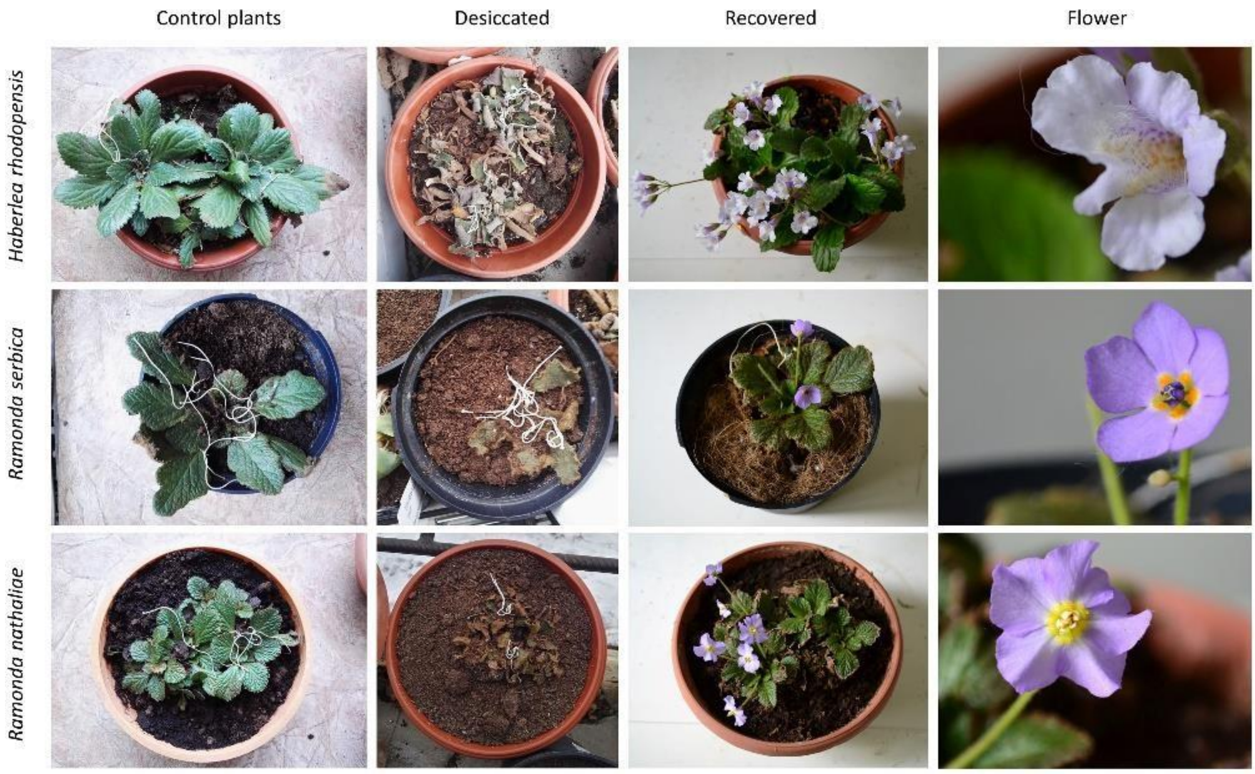

Figure S1. Control, desiccated by freezing temperatures, recovered plants as well as close-up view of *H. rhodopensis*, *R. serbica* and *R. nathaliae* flowers.

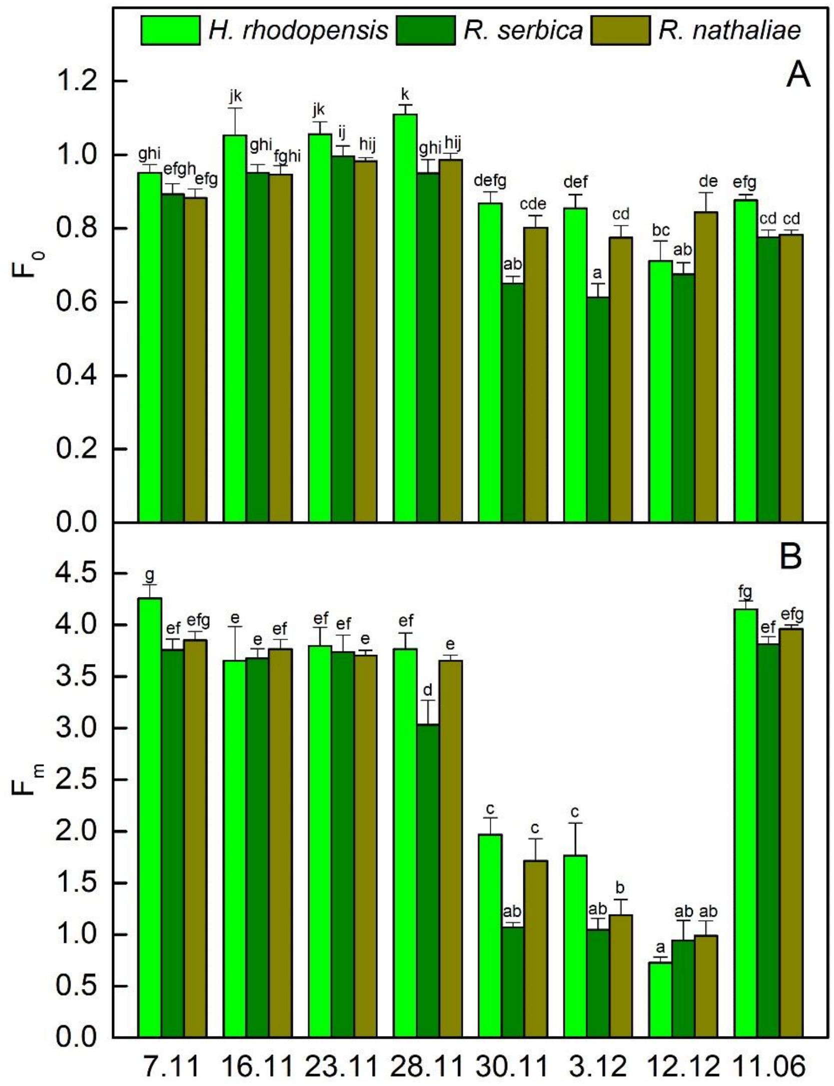

Figure S2. Minimum (F_0; A) and maximum (F_m; B) fluorescence levels of *H. rhodopensis*, *R. serbica* and *R. nathaliae* leaves during cold acclimation (7–28 November), freezing stress (30 November), freezing-induced desiccation (3 December–07 February) as well after recovery of plants (11 June) in ex situ environmental conditions. Values are given as mean ± SE. Data represent the mean of n = 6. Changes between plants were statistically compared. The same letters within a graph indicate no significant differences assessed by the Fisher LSD test ($p \leq 0.05$) after performing ANOVA.

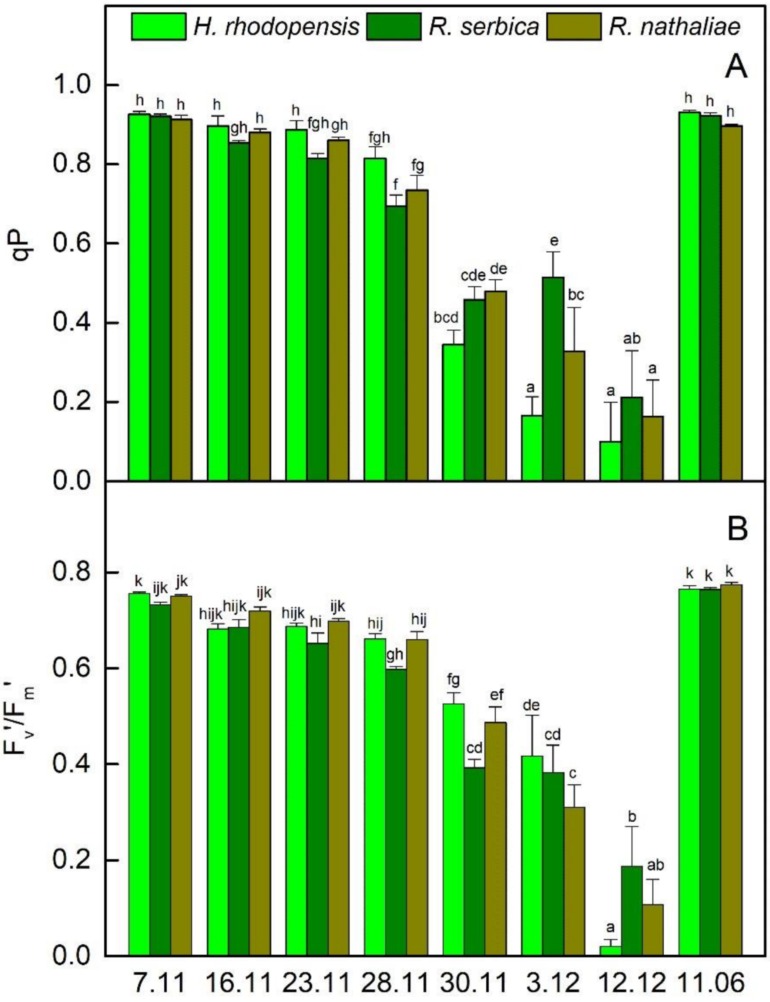

Figure S3. Changes of the open PSII centers, qP (A) and thermal energy dissipation, 1 – F_v'/F_m' (B) of *H. rhodopensis*, *R. serbica* and *R. nathaliae* leaves during cold acclimation (7–28 November), freezing stress (30 November), freezing-induced desiccation (3 December–07 February) as well after recovery of plants (11 June) in ex situ environmental conditions. Values are given as mean ± SE. Data represent the mean of $n = 6$. Changes between plants were statistically compared. The same letters within a graph indicate no significant differences assessed by the Fisher LSD test ($p \leq 0.05$) after performing.

ANOVA.
